# *N*-*acetyltransferase 2 *(*NAT2*) gene polymorphisms in Parkinson's disease

**DOI:** 10.1186/1471-2350-7-30

**Published:** 2006-03-29

**Authors:** Juergen Borlak, Stella Marie Reamon-Buettner

**Affiliations:** 1 Drug Research and Medical Biotechnology, Fraunhofer Institute of Toxicology and Experimental Medicine, Nikolai-Fuchs-Strasse 1, 30625 Hannover, Germany; 2 Chair in Pharmaco-and Toxicogenomics, Center of Pharmacology and Toxicology, Medical School Hannover, Carl-Neuberg-Strasse 1, 30625 Hannover, Germany

## Abstract

**Background:**

Parkinson's disease (PD) is a movement disorder caused by the degeneration of dopaminergic neurons in the substantia nigra of the midbrain. The molecular basis of this neural death is unknown, but genetic predisposition and environmental factors may cause the disease. Sequence variations in *N-acetyltransferase 2 *(*NAT2*) gene leading to slow acetylation process have been associated with PD, but results are contradictory.

**Methods:**

We analyzed three *NAT2 *genetic variations, c.481C>T, c.590G>A (p.R197Q) and c.857G>A (p.G286E), which are known to result in a slow acetylator phenotype. Using validated PCR-RFLP assays, we genotyped 243 healthy unrelated Caucasian control subjects and 124 PD patients for these genetic variations. Further, we have undertaken a systematic review of *NAT2 *studies on PD and we incorporated our results in a meta-analysis consisting of 10 studies, 1,206 PD patients and 1,619 control subjects.

**Results:**

Overall, we did not find significant differences in polymorphic acetylation genotypes in PD and control subjects. In the meta-analysis of slow acetylators from 10 studies and representing 604/1206 PD vs. 732/1619 control subjects, a marginally significant odds ratio (OR) of 1.32 (95% CI 1.12–1.54, p < 0.05) was obtained. Re-analysis of the data to exclude the only two studies showing positive association of slow acetylators to PD, resulted in a non-significant OR (1.07, 95% CI 0.9–1.28). Furthermore, meta-analysis of studies for c.590G>A, where both allele and genotype frequencies in PD vs. control subjects were analyzed, did not give significant summary odds ratios as well.

**Conclusion:**

We found little evidence for differences in polymorphic acetylation genotypes in PD and control subjects. Results of the meta-analyses did not also provide conclusive evidence for an overall association of *NAT2 *slow acetylator genotypes to PD.

## Background

Parkinson's disease (PD, MIM #16860) is a progressive neurodegenerative disorder characterized by resting tremor, muscular rigidity, bradykinesia (slowness of movement), postural instability, and pathologically, the presence of Lewy bodies. This movement disorder is caused by a deficiency of the neurotransmitter dopamine in the striatum of the brain, as a result of degenerating nigrostriatal dopamine neurons. The molecular basis of this neural death is unknown, but may be caused by genetic predisposition and gene environmental interactions, including exposure to pesticides, herbicides and neurotoxins [1]. For instance, rare pathogenic mutations and gene interactions in *α-synuclein*, *parkin *and *ubiquitin carboxy-terminal hydrolase LI (UCHL1) *have been implicated in PD [2]. A collaborative pooled analysis of 11 published studies of the *UCHL1 *p.S18Y variant involving 1,970 PD cases and 2,224 unrelated controls, confirmed an inverse association between this variant and PD particularly in younger subjects [3]. Furthermore, neurotoxins such as 1-methyl-4-phenyl-1,2,3,6-tetrahydropyridine (MPTP) and amine-related neurotoxins are proposed to be among the environmental factors, killing selectively dopaminergic neurons over a long period of time [4].

The *N*-acetyltransferases (NAT; E.C.2.3.1.5) are involved in the metabolism of drugs and environmental toxins. They catalyze the acetyltransfer from acetylcoenzyme A to an aromatic amine, heterocyclic amine or hydrazine compound. Sequence variations in the human *NAT1 *(MIM# 108345) and *NAT2 *(MIM # 243400) result in the production of NAT proteins with variable enzyme activity or stability, leading to slow or rapid acetylation. Therefore, genetic polymorphisms in *NAT1 *and *NAT2 *have been associated with drug-induced toxicities and disease (see reviews [5-7]).

Several association studies have been undertaken on *NAT2 *sequence variations and Parkinson's disease, but results are conflicting [8-20]. Some of these, report on strong associations between slow acetylator genotypes of *NAT2 *with PD in general or with early onset PD [8-13,18]. Slow acetylation may lead to insufficient detoxification of neurotoxins inducing PD, but the mechanism remains speculative. Recently, a study showed that intrastriatal 6-hydroxydopamine (6-OHDA) treatment of NAT2 slow acetylator rats led to decrease of striatal dopamine levels as compared to identical treatment of NAT2 rapid acetylator [21]. Intrastriatal 6-OHDA treatment in rats results in progressive dopaminergic nigral cell loss and constitutes an animal model for PD. To help clarify further the role of *NAT2 *acetylation genotypes in PD, we determined the *NAT2 *genotypes in unrelated PD patients and control healthy individuals of Caucasian origin focusing on three sequence variations, namely c.481C>T, c.590G>A, c.857G>A. We have also undertaken a systematic review of *NAT2 *studies on PD and we incorporated our present results in a meta-analysis consisting of 10 studies, 1,206 PD patients and 1,619 control subjects.

.

## Methods

Informed consent blood samples were kindly provided by Dr. C.A.D Smith of the Imperial Cancer Research Fund Laboratory of the Ninewell's Hospital in Dundee, U.K. In addition, JB has obtained approval to conduct genetic studies involving human materials from the Medical School of Hannover. All blood samples were obtained from randomly selected, unrelated Caucasian individuals. Using standard PCR-RFLP assay protocols, we employed the restriction enzymes *Kpn*I, *Taq*I and *Bam*HI to distinguish *NAT2 *variations c.481C>T (p.161L, dbSNP rs1799929), c.590G>A (p.R197Q, dbSNP rs1799930) and c.857G>A (p.G286E, dbSNP rs1799931), respectively. On the basis of these *NAT2 *variations, we genotyped 243 healthy unrelated Caucasian control subjects and 124 patients with Parkinson's disease (PD). The reference allele (NAT2*4) contains all three restriction sites, thus the identification of homo- and heterozygous carriers could easily be ascertained. In accordance with the human *NAT2 *nomenclature , allele NAT2*4 refers to *NAT2 *reference sequence (Genbank accession X14672). The NAT2*4 allele acts dominantly to result in rapid acetylation, and the presence of c.481C>T, c.590G>A, c.857G>A would lead to slow acetylation [22]. Therefore, for the determination of acetylator status, we classified those possessing at least two mutant alleles as slow acetylators. However, the acetylation status for the synonymous variation c.481C>T is not clear as this is also associated with allele NAT2*12C which is actually a rapid allele [7].

The confidence intervals for the percentages in our original study were computed according to Pearson-Clopper, whereas confidence intervals for the odds ratios were computed assuming asymptotic log normality. Meta-analysis was performed using the package rmeta of the R-Project [23]. The Mantel-Haenszel procedure meta.MH was applied to analyze the odds ratios of the studies, the Mantel-Haenszel summary, and Woolf's test for heterogeneity. The 95 % confidence intervals were also calculated for the individual and the summary odds ratios.

## Results

### Own study

We genotyped three *NAT2 *sequence variations (c.481C>T, c.590G>A and c.857G>A) in 243 healthy unrelated Caucasian control subjects and 124 PD patients. Although the difference in allele frequencies between control and PD was not statistically significant, we observed higher frequencies for NAT2*4 [c.481C + c.590G + c.857G] and c.857G>A, and lesser frequencies for c.481C>T and c.590G>A in PD (Table 1). Sequence variations in PD and control did not deviate from a Hardy-Weinberg equilibrium.

**Table 1:** Comparison of *NAT2 *allele frequencies in controls and Parkinson's diseased patients

Allele	Genetic variations			Control group (*n *= 243)	Parkinson's disease (*n *= 124)
	c.481C>T	c.590G>A	c.857G>A		
NAT2*4	C	G	G	22.3 %	28.9 %
*C*^*481*^*T *_*a*_	**T**	G	G	48.9 %	47.3 %
*G*^*590*^*A *_*b*_	C	**A**	G	27.0 %	21.5 %
*G*^*857*^*A *_*c*_	C	G	**A**	1.7 %	2.3 %

a) *C*^*481*^*T *= NAT2*5A, *5B, *5F, *5G,*5H, *5I, *6E, *11A, *11B, *12C *14C

b) *G*^*590*^*A *= NAT2*5E, *5J, *6A, *6B, *6C, *6D, * 6E, *14 D

c) *G*^*857*^*A *= NAT2*7A, *7B

The genotype frequencies are shown in Table 2. Heterozygotes for c.481C>T in control and PD did not vary much, but there tend to be more heterozygous carriers for c.590G>A in PD (Table 2). Moreover, while homozygotes for c.481C>T did not differ in control and PD, homozygotes for c.590G>A were more frequent in control. A homozygote for c.857G>A was only detected in PD. Further comparison of genotype frequencies indicated a two-fold increase of [c.590GA + c.857GA] and approximately one third reduction of [c.481CT + c.590GA] carriers in PD. The [c.857GA] genotype was only found in control population, but this represents only one of 243 individuals. Interestingly, 10 of 243 control individuals carried the two variations, [c.590AA + c.857GA], but the distribution of this genotype did not differ between control and PD. Overall, there was little evidence for statistically different distributions of genotypes in PD and control.

**Table 2:** *NAT2 *genotypes in controls and patients with Parkinson's disease

Genotypes			Deduced phenotypes	Controls		Parkinson's disease		Odds ratios (95% confidence interval)	χ^2^
				
c.481 C>T	c.590 G>A	c.857 G>A		*n *= 243	95% CI	*n *= 124	95% CI		
CC	GG	GG	rapid	5.4 % (13)	2.9 – 9.0	9.7 % (12)	5.1 – 16.3	1.90 (0.85–4.25)	NS
CT			rapid	23.0 % (56)	17.9–28.9	25.0 % (31)	17.7 – 33.6	1.11 (0.67–1.84)	NS
	GA		rapid	8.6 % (21)	5.4 – 12.9	15.3 % (19)	9.5 – 22.9	1.91 (0.99–3.68)	p < 0.01
		GA	rapid	0.4 % (1)	0.01 – 2.3	ND	0.0 – 2.9	0.000*	NS
TT			slow^1^	22.2 % (54)	17.2 – 28.0	20.2 % (25)	13.5 – 28.3	0.88 (0.52–1.51)	NS
	AA		slow	9.5 % (23)	6.1 – 13.9	5.7 % (7)	2.3 – 11.3	0.57 (0.24–1.36)	NS
	AA	GA	slow	4.1 % (10)	2.0 – 7.4	3.2 % (4)	0.9 – 8.1	0.78 (0.24–2.53)	NS
CT	GA		slow	23.9 % (58)	18.7 – 29.7	17.0 % (21)	10.8 – 24.7	0.65 (0.37–1.13)	NS
CT		GA	slow	2.5 % (6)	0.9 – 5.3	2.4 % (3)	0.5 – 6.9	0.98 (0.24–3.99)	NS
	GA	GA	slow	0.4 % (1)	0.01 – 2.3	0.8 % (1)	0.02 – 4.4	1.97 (0.13–30.24)	NS
		AA	slow	ND	0.0 – 1.5	0.8 % (1)	0.02 – 4.4	*	NS

^*^The confidence intervals for the percentages were computed according to Pearson-Clopper, whereas confidence intervals for the odds ratios were computed assuming asymptotic log normality. To avoid undefined results, some estimates were not computed.

^1 ^c.481 C>T is a silent mutation associated also with NAT2*12C (c.481 C>T, c.803A>G) and which is a rapid allele (see Hein et al. [7] )

### Meta-analysis

We found 13 original published association studies dealing with *NAT2 *gene polymorphisms in Parkinson's disease (see Background). Several studies were undertaken on white Caucasians, but there were also studies on Nigerian Africans [16], Hongkong Chinese [12], and Indians [13]. From these studies, a total of 1,961 cases and 2,433 control subjects have been analyzed for disease association between PD and *NAT2 *genetic variations. If we include our own study cohorts, the overall total would add up to 2,085 PD cases and 2,676 control subjects.

We summarized published information concerning characteristics of PD patients and control subjects (Table 3) as well as genetic and disease association analyses (Table 4) in 11 studies carried out from 1998–2005. Studies consisted of one 100% familial, three familial/sporadic and seven sporadic cases, and were either population or hospital-based. Average % male in PD cases was 59% (34–74%), while in control 53% (35–75%); average age at onset (AAO) was 64 (32–77 yr). Three studies stratified AAO into early onset (EOPD, <40 or <50 yr) and late onset (LOPD, >40 or >50 yr). Average age of exam (AAE) for PD cases was available for three studies (average 69 yr) and for control in 10 studies (average 63 yr, range 34–74). PD cases were diagnosed through manifestation of >2 cardinal features of PD (e.g. resting tremor, bradykinesia, postural disturbances, absence of and no apparent cause of parkinsonism, and/ or positive response to levodopa therapy). Control subjects were healthy family members, or randomly recruited healthy individuals from the same region, or in one case, pathologically normal brains from a brain bank. In two studies, control subjects were age-matched and in one study, 5–10 years older than the PD cases.

**Table 3:** Characteristics of studies on *NAT2 *polymorphisms and Parkinson's disease: patients and control subjects

Authors, country	Ethnicity of participants	**PD**						**Control**			
		
		Source	Type of PD cases	*N*	Male sex %	Age at onset (yr)	Diagnostic criteria	Source	*N*	Age at exam (yr)	Male sex %
Van der Walt et al. 2003, USA	NG^1^	DCHG Morris Udall PD Cntr., DCHG/Glaxo-Smith Kline PD Genetics Collaboration	familial	397 families 607 affected indvls.	59	62 ± 13 EOPD: ≤ 40 LOPD:> 40 AAE: 67 ± 12	levodopa therapy, absence of parkin mutations	healthy members of affected families	872 indvls.	67 ± 12	45
Bandmann et al. 1997, 2000, UK	Caucasian	UK PD Brain Bank, Inst. Psychiatry, London	familial, sporadic	100 familial 100 sporadic	55 familial 65 sporadic	68.4 ± 7.7 (51–85) age at death (sporadic): 76.7 ± 7.9 (51–94)	criteria, Maraganore et al. 1991^2^	UK PD Brain Bank, normal brains	100	77.1 ± 8.8 (60–90)	65
Nicholl et al. 1999, UK	Caucasian	West Midlands Region, UK	familial, sporadic	30 familial 176 sporadic	57 familial 60 sporadic	63.9 familial 64.5 sporadic	response to levodopa or dopamine agonists, = 2 cardinal PD features	hospitals in two separate regions within the UK West Midlands	30 familial 176 sporadic	63.9 familial 63 sporadic	57 familial 60 sporadic
Chaudhary et al. 2005, India	Indian	two hospitals: New Delhi, Bangalore	sporadic	267	74 EOPD: 69 LOPD: 79	31.8 ± 8.5 EOPD : ≤ 40 52.35 ± 9.0 LOPD:> 40	criteria, UK PD Brain Bank	healthy spouses of PD subjects, outpatient dept participating hospitals	324	45.83 ± 2.88 EOPD control 64.04 ± 8.13 LOPD control	75 EOPD control 74 LOPD control
Chan et al. 2003, China	Hongkong Chinese	two hospitals, Hongkong	sporadic	99	60	71.7 (46–93) 4 patients ≤ 45	criteria, Maraganore et al. 1991^2^, levodopa therapy	same hospitals as PD cases	126	70.2 (39–96)	50
Bialecka et al. 2002, Poland	Caucasian	Pomeranian region	sporadic	54	NG^1^	68	= 2 cardinal PD features	same region, randomly selected	85	73.4 ± 5.8(65–87)	NG^1^
Maraganore et al. 2000, USA	Caucasian (almost)	Mayo Clinic referrals (from MN, WI, ND, SD) and PD study Olmsted County, MN	sporadic	139	65	62 (31–82) AAE: 69 (39–91)	= 2 cardinal PD features, levodopa therapy	Olmsted County, MN, healthy spouses of PD subjects	113	72 (37–90)	35
Dupret et al. 1999, France	Caucasian	hospitals and clinics in Champagne-Ardenne	sporadic	68	67	61 ± 9 (25–88) AAE: 71 ± 9	cardinal PD features	same region as PD cases	211	43.8 ± 6.1	50
Harhangi et al. 1999, Netherlands	Caucasian	PD cases in Rotterdam	sporadic	80	34	77.3 ± 8.3 (57–99)	= 2 cardinal PD features	same region as PD cases	161	78 ± 8.3 (57–98)	39
Agundez et al. 1998, Spain	Caucasian	hospitals in Madrid and vicinity	sporadic	121	EOPD: 54 LOPD: 52	41.3 ± 6.4 (28–50) OEPD: 28–50 67.3 ± 8.2 (51–83) LOPD : >51	criteria, Hughes et al. 1992^3^	volunteers from the same region	121	34.2 ± 12.5	53

^1^NG  =not given

^ 2^ Maraganore DM, Harding AE, Marsden CD. A clinical and genetic study of familial Parkinson's disease. Mov Disord 1991; 6:205-211.

^3 ^Hughes AJ, Daniel SE, Kilford L, Lees AJ. Accuracy of clinical diagnosis of idiopathic Parkinson's disease: a clinico-pathological study of 100 cases. J Neurol Neurosurg Psychiatry 1992; 55:181-184.

**Table 4:** Characteristics of studies on *NAT2 *polymorphisms and Parkinson's disease: genetic and disease-association analyses

Authors, country	Samples	*NAT2 *variants	Assay	Statistical analysis	Slow acetylator PD cases (%)	Slow acetylator controls (%)	Association Yes/No
Van der Walt et al. 2003, USA	blood	282, 341,481,590, 857	oligo ligation assay	pedigree disequilibrium test (PDT-sum), likelihood ratio test (Transmit), χ^2^-test	NG^1^	NG^1^	No overall, but marginal c.282 in EOPD
Bandmann et al. 2000, UK	blood, brain tissue	191, 341,282,481,590, 803, 857	allele-specific CR, PCR-RFLP	χ^2^-test, OR 95% CI, Yate's correction	familial: 69.0 sporadic: 59.0	37.0	Yes
Bandmann et al. 1997, UK	blood, brain tissue	481, 590, 857	PCR-RFLP	χ^2^-test, OR 95% CI, Yate's correction	familial: 73 sporadic: 60	39.0	Yes
Nicholl et al. 1999, UK	blood	481, 282, 590, 803, few cases: 191, 857	PCR-RFLP	χ^2^-test, two-sample *t- *or Wilcoxon tests, OR 95% CI	familial: 53.3 sporadic: 60.1	familial: 76.7 sporadic: 55.4	No
Chaudhary et al. 2005, India	blood	191, 282, 341,481, 590, 803, 857	PCR-RFLP	χ^2^-test, PHASE 2.0.2 for haplotypes, OR 95% CI	pooled: 19.2 EOPD: 25.78 LOPD: 12.29	pooled: 13.45 EOPD control: 13.01 LOPD control: 13.78	Yes, c.590 and c.857 in EOPD c.282 in LOPD
Chan et al. 2003, China	blood	481,590,857	PCR-RFLP	logistic regression analysis, adjusted OR 95% CI	68.7	28.6	Yes
Bialecka et al. 2002, Poland	blood	481,590,803,857	PCR-RFLP	χ^2^-test, OR 95% CI, Yate's correction	64.8	46.9	Yes
Maraganore et al. 2000, USA	blood	481,590, 857	PCR-RFLP	logistic regression analysis, adjusted OR 95% CI, two-sided *p *values	54.0	53.0	No overall, but c.857
Dupret et al. 1999, France	blood	191,341,590,857	allele-specific PCR, PCR-RFLP	NG^1^	51.5	59.8	No
Harhangi et al. 1999, Netherlands	blood	481,590, 857	PCR-RFLP	OR 95% CI, Fischer's exact test two tailed	54.0	53.0	No
Agundez et al. 1998, Spain	blood	191, 282, 341, 590, 803, 857	PCR-RFLP	χ^2^-test, OR 95% CI, Fischer's exact test	EOPD: 78.4 LOPD: 54.8	55.4	No overall, but yes OEPD

NG^1^=not given

Genomic DNA from blood samples was used in all studies but one, and PCR-RFLP was mainly the method employed for genotyping analysis. All studies basically analyzed the *NAT2 *variants c.481C>T, 590G>A, 857G>A and in all, the variant c.590G>A. Disease association was determined generally by computing odds ratios, except for a family-based study in which pedigree disequilibrium (PDT) or likelihood ratio (Transmit) tests were used [20]. Either there was overall association of slow acetylator genotypes to PD [9,10,12], or there was no overall association [8,14,15,18-20]. In addition, two earlier studies did not also find association between slow acetylator genotypes and PD [16,17]. Some studies, however, found positive association of certain variants (e.g. c.857G>A, [18], in particular to early-onset PD [8,13].

**Figure 1:**
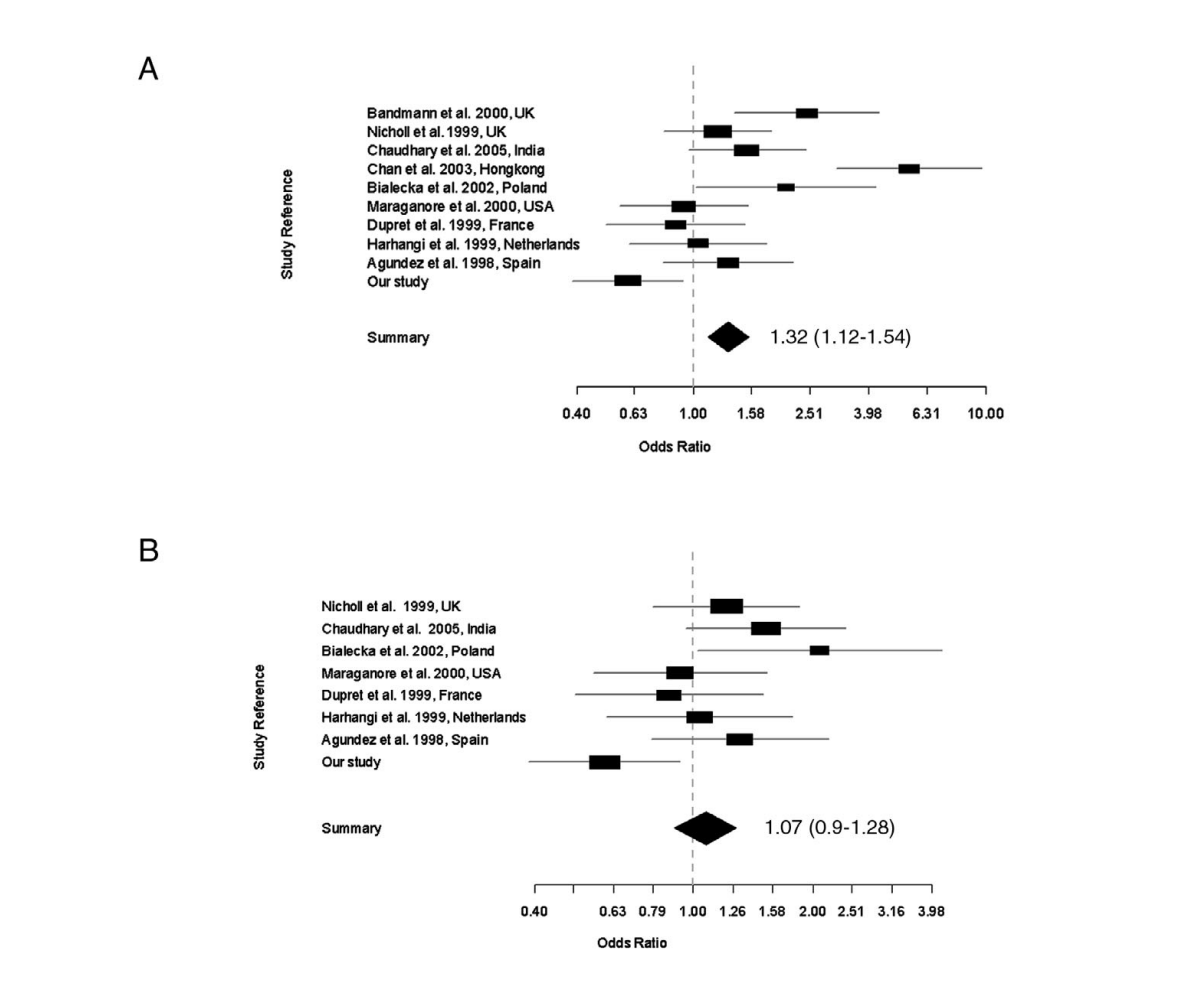
**Meta-analysis of association studies on *NAT2 *slow acetylators and sporadic Parkinson's disease. **The confidence interval for each study is given by a horizontal line, and the point estimate is given by a square whose height is inversely proportional to the standard error of the estimate. The summary odds ratio is drawn as a diamond with horizontal limits at the confidence limits and width inversely proportional to its standard error. **(A)**: Meta-analysis on 10 studies. Test for heterogeneity χ^2 ^(df 9) = 47.71 (p-value 0). The odds ratios are significantly different between the studies. The summary odds ratio (Mantel-Haenszel OR= 1.32, 95% CI, 1.12–1.54) is significantly greater than 1 (p < 0.05). **(B) **Meta-analysis on 8 studies, excluding studies of Bandmann et al. 2000 and Chan et al. 2003. The odds ratios are not significantly different between studies. Test for heterogeneity χ^2 ^(df 7) = 14.28 (p = 0.0464). The summary odds ratio (OR= 1.07, 95% CI, 0.9–1.28) is not significant.

To obtain synthesis of data from multiple studies, we have undertaken a meta-analysis of slow acetylators in PD vs. control subjects in 10 studies including our own (Fig. 1A). Only data on sporadic cases were included. The odds ratios were significantly different between the studies (p = 2.89334e-07), and were consistent with respective authors' conclusions. For the pooled analysis of 604/1206 PD vs. 732/1619 control subjects, we obtained a Mantel-Haenszel OR of 1.32 (95% CI 1.12–1.54) which was significantly greater than 1 (p < 0.05). Because the studies of Bandmann et al. 2000 [10], and Chan et al. 2003 [12] were the only ones showing positive association of slow acetylators to PD, excluding these investigations resulted in a non-significant OR (1.07, 95% CI 0.9–1.28) (Fig. 1B). Our own study appeared to show an inverse association of slow acetylators to PD (OR 0.60, 95% CI 0.39–0.93, based on 62/124 PD vs.152/243 controls), but the ORs in Fig. 1B did not differ significantly between each other.

**Figure 2:**
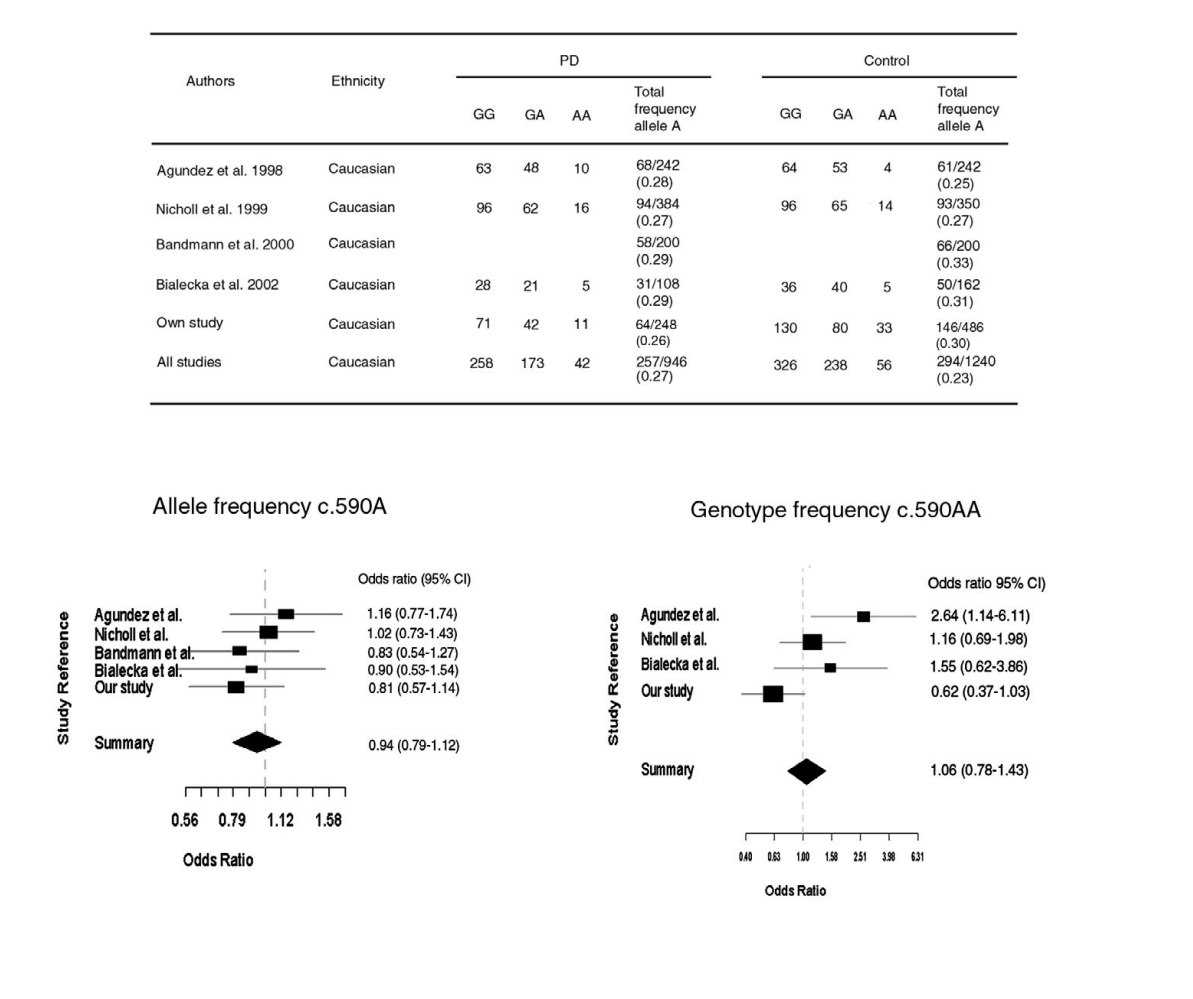
**Meta-analysis of *NAT2 *c.590G>A in PD and control subjects. **The confidence interval for each study is given by a horizontal line, and the point estimate is given by a square whose height is inversely proportional to the standard error of the estimate. The summary odds ratio is drawn as a diamond with horizontal limits at the confidence limits and width inversely proportional to its standard error. Allele frequency: Test for heterogeneity χ^2 ^(df 4) = 2.36 (p-value 0.67). The odds ratios are not significantly different between the studies (p = 0.67). The summary odds ratio (Mantel-Haenszel OR= 0.94, 95% CI, 0.79–1.12) is not significantly greater than 1 (p > 0.05). Genotype frequency: Test for heterogeneity χ^2 ^(df 3) = 9.58 (p-value 0.0225). The odds ratios are not significantly different between the studies (p = 0.0225). The summary odds ratio (Mantel-Haenszel OR= 0.94, 95% CI, 0.78–1.43) not significantly different from 1.

Furthermore, our analysis of five studies for c.590G>A comparing the allele frequency A in PD vs. control subjects found that odds ratios were not significantly different between the studies (p = 0.67) (Fig. 2). The summary OR= 0.94 (95% CI, 0.79–1.12) did not deviate significantly from 1. Similarly, analysis of four studies for the genotype c.590AA did not find significant differences between studies, and the summary OR = 1.06 (95% CI, 0.78–1.43) was likewise not significant (Fig. 2).

## Discussion

Overall, we demonstrate little evidence for differences in polymorphic acetylation genotypes when a control and Parkinson's diseased population is compared. This finding may contrasts previous studies, but is similar to results of other studies on Caucasians where no overall association has been found (see Meta-analysis, Results). For instance, one study found a strong association between slow acetylator genotypes and familial PD (*n *= 100) in European patients [10]. Another study found a statistically significant predominance of slow acetylators in sporadic PD (*n *= 54) in Polish population [11]. In unrelated Hongkong Chinese with PD (*n *= 99), a strong association between *NAT2 *genotypes and PD was likewise found [12]. Recently, in an Indian population, a significant association was found for c.590G>A and 857G>A in early-onset, as well as c.282C>T in late-onset PD [13].

Nonetheless, there are also studies which do not show association between *NAT2 *genotypes and PD. For instance, no statistically significant differences were found after comparison of *NAT2 *genotypes between sporadic PD (*n *= 121) and control group [8]. They observed, however, that patients with early-onset PD (before 50 years, *n *= 37), displayed a higher frequency of slow acetylator types. Further, Dupret et al. [14] found similar distribution of slow acetylators in sporadic French PD (*n *= 68) and control. Harhangi et al. [15] found likewise that overall frequencies were distributed similarly among PD (*n *= 132) patients and control. Of considerable importance is the finding of a recent family-based study which analyzed 397 families (*n *= 1580 individuals, 607 affected) and found no overall association between any *NAT2 *genetic variations (single or combined) and familial or early-onset PD [20].

Our meta-analysis of 10 studies of slow acetylators representing data from 1,206 PD patients and 1,619 control subjects gave an OR value of 1.32 (95% CI 1.12–1.54) which was marginally significant (p < 0.05). This result is similar to an earlier meta-analysis where a total of 792 PD patients and 912 control subjects from seven studies published between 1989–1999 were investigated. Specifically, the frequency of slow to rapid acetylator genotypes for the composite seven studies showed a significant association of patients with PD to control subjects (OR = 1.33; 95% CI, 1.08 to 1.62) [23]. However, our meta-analysis on eight studies, omitting the studies of Bandmann et al. 2000 [10] and Chan et al. 2003 [12], resulted in an OR of 1.07, 95% CI 0.9–1.28, which was not significant. This result shows that these two studies were solely responsible for the marginal positive association obtained in the meta-analysis of 10 studies. Indeed, the inclusion of Chan et al. 2000 [12] in a meta-analysis of nine studies would still give an OR of 1.25, 95% CI, 1.06–1.47, and p <0.05. Although our study appeared to show an inverse association of slow acetylators to PD (OR 0.60, 95% CI 0.39–0.93), this OR did not differ significantly from other studies (see Fig. 1B).

Furthermore, our analysis of five studies for c.590G>A comparing the allele frequency A in 473 PD vs. 620 control subjects found that the summary OR 0.94 (95% CI, 0.79–1.12) did not deviate significantly from 1. Similarly, the odds ratios in four studies for the genotype c.590AA was not different from each other and the obtained OR value (OR = 1.06, 95% CI 0.78–1.43) was not significantly different from 1. Therefore, there appears to be no overall association of slow acetylator genotypes to PD.

Our systematic review and meta-analysis of *NAT2 *studies on PD could serve as an initial assessment or synthesis of studies towards understanding of the specific role of *NAT2 *acetylation variants in PD. As reflected in the summary of *NAT2 *studies and PD (see Tables 3 and 4), there is lack of uniformity in study design as well as in the genetic analysis and interpretation of results. Such difficulties and confusions regarding *NAT2 *studies and PD have been outlined earlier [24]. For instance, in some studies there was no report on genotype frequencies or studies have different focal emphasis, i.e. familial or sporadic PD. To determine specifically the role of *NAT2 *acetylation variants in PD, perhaps a collaborative effort is needed with uniform study design involving different ethnic groups as has been demonstrated in the meta-analysis of *UCHL1 *studies in PD [3].

## Conclusion

Overall, our study did not find significant differences in polymorphic acetylation genotypes in PD and control subjects. The meta-analysis on 10 studies showed a marginal positive association of slow acetylators to PD, but this result was driven by two studies, the exclusion of which in the re-analysis provided a non-significant estimate. Apparently, there appears to be no overall association of *NAT2 *slow acetylator genotypes to Parkinson's disease.

## Competing interests

The author(s) declare that they have no competing interests.

## Authors' contributions

JB was responsible for the conception and design, acquisition and analysis of data, interpretation of results and writing of the manuscript, and have given final approval of the version to be published. SMRB participated in the conception and design, acquisition and analysis of data, interpretation of results and writing of the manuscript.

## Pre-publication history

The pre-publication history for this paper can be accessed here:


